# Multiple arterial grafting and ostriches: let’s all take heart!

**DOI:** 10.18632/oncotarget.21392

**Published:** 2017-09-30

**Authors:** Antonino Di Franco, Filippo M. Sarullo, Mario Gaudino

**Affiliations:** Mario Gaudino: Department of Cardiothoracic Surgery, Weill Cornell Medicine, New York, NY, USA; Cardiovascular Rehabilitation Unit, Buccheri La Ferla Fatebenefratelli Hospital, Palermo, Italy

**Keywords:** CABG, total arterial revascularization, third arterial graft, arterial revascularization, multiple arterial grafting

The history of surgical revascularization with arterial conduits has followed an intriguing evolution. Starting from the seminal paper by Loop et al.[1] which resulted in the widespread introduction of the left internal thoracic artery (LITA), evidence has been accumulated supporting the use of a second arterial conduit, despite initial skepticism mainly concerning the risk of deep sternal wound infections. Over time, it has become evident that the benefits of using a second arterial conduit not only are related to better graft patency, but also extend to better long-term survival, even in higher-risk cohorts. Nonetheless, despite the large amount of available evidence, the adoption rate of arterial conduits in coronary artery bypass (CABG) patients still remains low. A recent paper from the Society of Thoracic Surgeons highlighted that a second arterial conduit is currently used in far less than 10% of cases with no increase in the last decade,[2] even though American guidelines recommend total arterial revascularization in young patients with reasonable life expectancy (Class IIb).[3]

Interestingly, no large randomized clinical trial on the topic has been completed so far; the 10-year results of the only ongoing large trial comparing the clinical outcomes of CABG patients receiving 1 *vs*. 2 ITAs (ART trial) are eagerly awaited (especially after the publication of the controversial 5-year interim analysis).[4] However, over the last 2 decades, a large number of observational studies and meta-analyses have shown a clear advantage of bilateral ITAs (BITA) over single ITA (SITA).

That being said, the next logical step would be to assess whether 3 arterial conduits could grant a survival benefit over 2. Up until now, only few authors have focused their attention on the potential advantages of a third arterial graft strategy over 2, with conflicting results. All of these studies not only come from single institutions, they are also limited by relatively small sample sizes, so that moderate differences in survival can hardly be detected. This is of particular concern in the peculiar case of the potential additional survival benefit of a third arterial conduit, which is expected to be less when compared to that of a second.

For all these reasons, we analyzed the current available evidences on the use of 3 versus 2 arterial conduits, using a meta-analytic approach based on propensity score matched studies, which is known to be the most reliable strategy when large randomized clinical trials are not possible or not available.[5] By investigating a total of 10,287 matched patients, the use of 3 arterial grafts was found to be associated with significantly lower hazard for late death (hazard ratio, 0.8; 95% confidence interval, 0.75–0.87; *P* < 0.001), irrespective of sex and diabetes mellitus status. This provides another important proof in favor of the use of multiple arterial conduits in CABG patients, adding to a growing body of literature demonstrating the advantages of total arterial strategy. Of note, these results have been recently confirmed by Yanagawa et al. [6], who performed a meta-analysis comparing the outcomes of total arterial revascularization (TAR) versus different conventional revascularization strategies. Once again, when compared to 2 arterial grafts, TAR was associated with reduced long-term all-cause mortality (incident rate ratio 0.85, 95% CI: 0.73–0.99, *p* = 0.04).

What are the reasons that limit adoption of total arterial revascularization then? Why would a surgeon still prefer SITA? Explanations are multiple and certainly complex. As elegantly noted by Rosengart,[7] in the current era of extremely favorable outcomes expectations for CABG, the fear of acute risks (e.g.: deep sternal wound infections) looms large, “*especially when compared with the relatively ephemeral promise of long-term benefit that may not materialize for approximately a decade after surgery*”. Long story short, the quest for “instant gratification” could have affected not only physicians’ attitude, but also reimbursement systems towards the choice of multiple arterial grafting, a strategy perceived to be more complex, time-consuming and potentially risky in the short-term.

Mere immediate convenience might drive decisions towards SITA adoption. Nevertheless, this implies that we deliberately ignore existing evidence at the cost of our patients’ long-term survival. One might argue that no sufficiently large randomized trial is so far available and no level A evidence for multiple arterial grafting can therefore be advocated. That is correct, and it looks like a very rigorous scientific approach to this debate. But at the same time… is the use of SITA supported by any large prospective randomized clinical trial? It is not. As Ramzy stressed out,[8] the above mentioned landmark study by Loop et al.[1] not only was single-center, but also retrospective, with all the drawbacks and weakness that these types of studies come with. Still, it effectively prompted the widespread adoption of LITA, which eventually became the gold standard for CABG surgery. Good decisions can be taken with “less than perfect” information. Of note, the body of evidence on multiple arterial grafting is broader than what was available in 1986 when the LITA strategy was presented by Loop. Yet, the vast majority of surgeons still neglect it.

Let’s all take heart! Multiple arterial revascularization era has unquestionably already started; ignoring this would only mean deliberately burying our head in the sand: nobody wants to be an ostrich.
